# Testing the mediating effect of risk perception on the relationship between e-health literacy and infectious disease prevention behaviors among young adults based on the IMB model

**DOI:** 10.3389/fpubh.2025.1728099

**Published:** 2025-12-11

**Authors:** Gye Hyun Jung, Hye Young Song

**Affiliations:** 1 Department of Nursing, Howon University, Gunsan-si, Republic of Korea; 2 Department of Nursing, Woosuk University, Wanju-Gun, Republic of Korea

**Keywords:** young adult, health literacy, risk perception, infection control, health behavior

## Abstract

**Background:**

This study aimed to investigate the influence of e-health literacy on infectious disease prevention behaviors among young adults in their 20s and 40s based on the information–motivation–behavioral skills model and to test the mediating effect of infectious disease risk perception on this relationship.

**Methods:**

This cross-sectional survey study included young adults aged 20–40 years recruited through anonymous snowball sampling using online communities and social media. Variables were measured using the Korean version of the K-eHealth Literacy Scale, infectious disease risk perception, and self-reported infectious disease prevention behaviors. Data were analyzed using descriptive statistics, *t*-tests, and Pearson’s correlation analysis, and the mediating effect of infectious disease risk perception was tested using Hayes’ PROCESS macro (Model 4).

**Results:**

The mean of the main variables in this study was 3.55 for e-health literacy, 3.09 for risk perception, and 3.99 for preventive behaviors. Correlation analysis showed that preventive behaviors were significantly positively related to e-health literacy (*r* = 0.22, *p* < 0.001) and risk perception (*r* = 0.40, *p* < 0.001), and that e-health literacy was significantly positively related to risk perception (*r* = 0.22, *p* < 0.001). Preventive behaviors differed significantly according to sex, employment status, diagnosis experience, and quarantine experience. In the regression analysis, e-health literacy had a significant effect on risk perception (*B* = 0.120, *p* = 0.005) and in its total effect on preventive behaviors (*B* = 0.103, *p* = 0.041). However, after controlling for risk perception, the direct effect of e-health literacy was not significant (*B* = 0.055, *p* = 0.255), and only risk perception had a significant effect on preventive behaviors (*B* = 0.401, *p* < 0.001). A significant indirect effect of e-health literacy on preventive behaviors was confirmed by the mediation analysis (*B* = 0.048, 95% confidence interval [0.016, 0.086]) and a full mediation effect of risk perception.

**Conclusion:**

e-health literacy education alone is insufficient, and interventions that combine accurate risk communication and perception enhancement are needed. Tailored programs that consider life contexts, such as the workplace, may be effective in improving preventive behaviors among young adults.

## 1 Introduction

Infectious disease prevention behaviors are essential for personal and public health. During the coronavirus disease (COVID-19) pandemic, preventive behaviors such as mask wearing, hand washing, and distancing were highly effective in reducing mortality and transmission (1); however, adherence rates among young adults in their 20s and 40s were reported to be lower than those among other age groups, even though they are more socially and economically active and may be a key link in the transmission of infectious diseases (1, 2). During the COVID-19 pandemic, frequent social contact among young adults has been a major factor contributing to community outbreaks (3), and as they are often asymptomatic or mildly infected due to their high immunity, they are unaware of their infection status and are less likely to follow instructions such as avoiding public facilities because of their confidence in their health (4, 5).

According to recent infectious disease outbreaks in Korea and abroad, the proportion of young adults in their 20s–30s is increasing significantly for various infectious diseases, such as measles, syphilis, tuberculosis, and human immunodeficiency virus (HIV). In 2025, the number of participants with measles in Korea increased 1.3 times year-on-year, and the main age group of infection was in their 20s (6). Additionally, the proportion of female participants is steadily increasing in the case of syphilis, a sexually transmitted disease (7). Tuberculosis and HIV infections also account for a high proportion of people in their 20s and 30s (8). Moreover, young adults have low rates of immunization against key vaccines as they progress to adulthood, leading to immunization gaps (9). As they are in a variety of socioeconomic settings, including academia, work, and irregular employment, quarantine or treatment of infectious diseases can lead to immediate economic losses, especially for young adults with employment insecurity (10). Moreover, as climate change and urbanization are expected to increase the frequency of future pandemics (11), national and international studies have focused on the determinants of preventive behaviors among young adults (4, 12). Therefore, in-depth research on young adults is needed to develop strategies that will lead to voluntary and sustained preventive behaviors in preparation for the next infection crisis.

The information–motivation–behavioral skills (IMB) model (13) is a theory for explaining and predicting health behaviors. It posits that behavioral change occurs when there is a combination of accurate information, sufficient motivation, and feasible behavioral skills. Recent research has identified the IMB model as an appropriate theoretical framework for understanding the risk of future pandemics and the determinants of preventive behaviors (11) and has been successfully applied to promote behavioral change in a variety of health domains (14, 15). In particular, young adults who are accustomed to digital environments are exposed to a vast amount of health information, but are also easily influenced by misinformation (16). It is therefore necessary to use the IMB model to integrate cognitive and emotional factors to predict their infectious disease prevention behaviors.

E-health literacy refers to the ability of individuals to locate, understand, evaluate, and act upon health information in a digital environment (17). Its characteristics are qualitatively different from those of traditional health information literacy, and it is becoming increasingly important during infectious disease crises such as pandemics (18). In particular, young adults are familiar with the digital environment and exposed to a vast amount of health information; however, they are also susceptible to the influence of misinformation (16). Digital information often has unclear origins and is mixed with false information; therefore, assessment and verification of its reliability are crucial (19, 20). This is because e-health literacy goes beyond simply “knowing” information; it includes the “ability to evaluate” information within networked environments (19).

According to previous research, individuals with high e-health literacy can filter out misinformation and recognize evidence-based information (21, 22), and they are more likely to accurately perceive infection risks and actively engage in preventive behaviors (23). Indeed, during the COVID-19 pandemic, a study among university students showed that e-health literacy and risk perception were significantly associated with preventive behaviors (24), and a study targeting healthcare workers also found that e-health literacy was related to adherence to infection prevention guidelines and lifestyle changes (22). The IMB model theoretically systematizes the complexity of the digital information environment and the new phenomenon of misinformation, offering unique value by effectively predicting and promoting digital health behaviors among young adults. It particularly clarifies the mechanism by which the information evaluation capability of e-health literacy translates into accurate risk perception and ultimately leads to the practice of preventive behaviors.

Studies have also reported that increased e-health literacy reduces fear and promotes health behaviors based on accurate information (12). As such, e-health literacy may be the key to enhancing risk perception and driving healthy behaviors (24).

Risk perception has also been identified as an important motivating factor in infectious disease prevention. Higher levels of subjectively perceived infection risk tend to be associated with higher levels of preventive behaviors (25). In a study of over 1,800 community adults in Iran, perceived risk of the COVID-19 pandemic significantly predicted adherence to preventive behaviors (handwashing and distancing) (26), and a study in China found that perceived risk, such as fear and anxiety, can motivate protective behaviors (27). A meta-analysis also reported that higher perceived severity increases vaccination intention (28), suggesting that it consistently promotes protective behavior.

**Figure 1:**
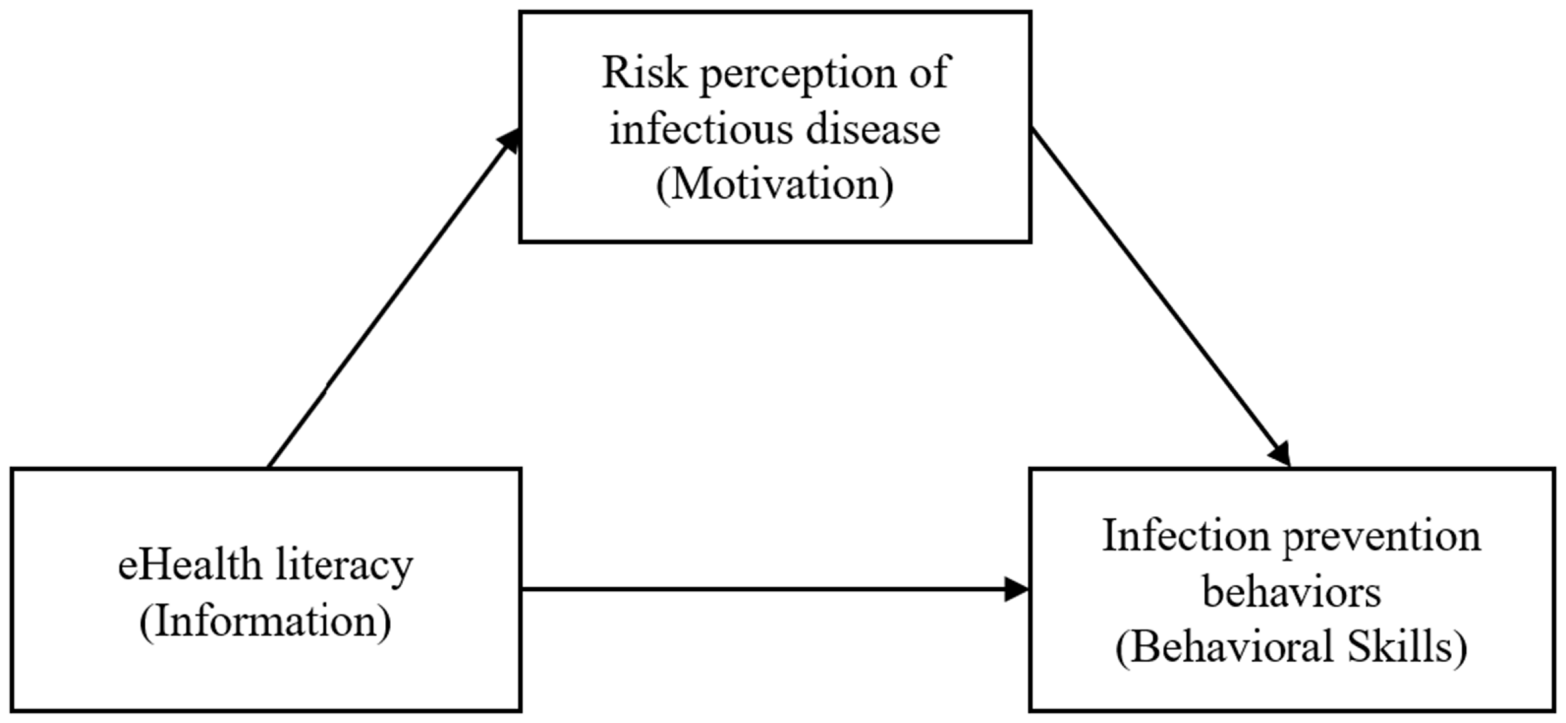
Framework.

Based on this evidence, this study analyzes the relationship between e-health literacy, which is a precise information component of the IMB model; risk perception (perceived risk of infection), which is a sufficient motivation component; and infectious disease prevention behavior, which is a behavioral component, in a structural model of health behavior among young adults (Figure 1). By identifying the influence pathways of e-health literacy and risk perception on preventive behaviors, this study will provide a basis for customized intervention strategies to promote infectious disease prevention behaviors among young adults and provide empirical evidence for the applicability of the IMB model.

### 1.1 Purpose of the study

This study aimed to investigate the impact of e-health literacy on infectious disease prevention behaviors among young adults in their 20s–40s using the IMB model as a theoretical framework and to test the mediating role of risk perception in this relationship.

## 2 Methods

### 2.1 Research design

This study used a cross-sectional survey based on the IMB model to test the effect of e-health literacy on infectious disease prevention behavior and the mediating effect of risk perception among young adults in their 20s and 40s.

The study design was grounded in the IMB model (13), which posits that health behavior change occurs through the integration of three core components: accurate information, sufficient motivation, and feasible behavioral skills. As illustrated in Figure 1, we operationalized these theoretical components as described below.

#### 2.1.1 Information component

E-health literacy was selected as the primary information variable, representing the ability of individuals to search for, understand, evaluate, and apply online health information (17). Given that young adults predominantly access health information through digital platforms (18), e-health literacy serves as a critical indicator of their capacity to process infection-related information.

#### 2.1.2 Motivation component

Risk perception of infectious disease was operationalized as the motivational factor, reflecting the subjective assessment of infection susceptibility and severity by individuals (25, 26). According to the IMB model, accurate information alone is insufficient to drive behavior change; individuals must also perceive personal relevance and risk to be motivated to act.

#### 2.1.3 Behavioral component

Infection prevention behaviors, including adherence to recommended practices such as hand hygiene, mask-wearing, physical distancing, and ventilation, were assessed as the behavioral outcome (29, 30).

On the basis of this theoretical framework, we tested the following hypotheses:

*H1*: E-health literacy positively affects infectious disease prevention behaviors among young adults.

*H2*: Risk perception mediates the relationship between e-health literacy and infectious disease prevention behaviors.

This aligns with recent applications of the IMB model to pandemic-related behaviors (11) and allows for systematic examination of the ways in which e-health literacy can influence preventive practices by enhancing risk perception.

### 2.2 Data collection and study participants

The study participants were young adults between the ages of 20 and 40. The data collection period was from August 27, 2025 to September 12, 2025, using a nonprobability snowball sampling method and was conducted in an anonymous, diffuse manner in accordance with the principle of minimal collection of personal information. Initial seed participants (approximately 10–15) were recruited through public announcements by the researchers (online communities, company communities, etc.) and were provided with a link to a public information page and a one-time invitation code that did not require personalization or real-name referrals. For initial seed and follow-up participants, we did not record or report who invited them, and they were encouraged to freely share the link with whomever they wished among young adults between the ages of 20 and 40. This approach ensured that no relationship information between inviter and recipient was collected or stored.

All responses were collected via an anonymous online survey (10-min intervals) using Google Forms (Google LLC) as the web-based data collection platform, and no personally identifiable information was collected. No personally identifiable information was collected, and a one-time invitation code was set up on the server to prevent duplicate responses. The electronic consent process was presented on the first screen. The purpose of the study, items collected (minimally), storage period, right to withdraw consent and data deletion, de-identification, and prohibition of third-party provision were explained in the pre-participation online information sheet, and the survey was started only after consent was obtained. Participants received a small compensation (a coffee voucher) upon completing the survey, which was provided uniformly to all respondents.

Sample sizes were calculated based on multiple regression analysis using the G*Power 3.1 program. With an effect size of *f*^2^ = 0.15, a significance level of 0.05, a power of 0.95, and 14 predictor variables (12 general characteristics and two main influencing variables), the minimum required sample size was 204, and 250 participants were finally recruited to account for the dropout rate. After excluding six nonrespondents, 224 questionnaires were included in the final analysis.

#### 2.2.1 Inclusion criteria

- Young adults aged 20–40 years who have lived in Korea for at least 6 months.
- Those who understood the study description in Korean agreed to provide electronic consent.
- Those who could independently complete a self-administered online survey on personal devices.
- The researcher distributed a link containing a one-time invitation code to those who accessed and submitted the survey only once.

#### 2.2.2 Exclusion criteria

- Those currently diagnosed with or under treatment for acute infectious diseases at the time of survey participation.
- Those with severe cognitive impairment or psychiatric conditions that would impair their ability to accurately respond to survey items.

### 2.3 Instruments

#### 2.3.1 General characteristics

Data on the sex, age, marital status, education level, occupation, household economic status, infectious disease prevention education experience, infectious disease-related information, subjective health status, infectious disease diagnosis experience, infectious disease isolation experience, and chronic disease status of household members were collected.

#### 2.3.2 Health literacy (e-health literacy scale)

Chung et al. (31) translated and validated K-eHEALS; a total of 10 items were used, of which eight (items 3–10) were used to calculate a score that reflects Internet health literacy, and each item was rated on a 5-point Likert scale. The total score ranged 8–40, with higher scores indicating higher Internet health literacy. Cronbach’s α in the original study was 0.88, and in the Korean version of the validation study (31), it was 0.89. In this study, Cronbach’s α was 0.94 (CFI > 0.90, TLI > 0.90, RMSEA < 0.08).

#### 2.3.3 Risk perception

The tool used to identify the risk perception of fine dust in the study by Lee and Kim (32) was used to identify the risk perception of four subfactors of emerging infectious diseases in the study by Park (33): economic perception, social perception, physical perception, and human relationships. The risk perception tool consisted of 12 questions, which were modified and supplemented to identify the risk perception of infectious diseases, and content validity was checked by one infectious disease nurse and two nursing professors. Each item is scored on a 5-point Likert scale ranging from 1 (not at all) to 5 (very much so), with higher scores indicating a higher risk perception. In Park et al.’s (29) study, Cronbach’s α = 0.730; in this study, Cronbach’s α was 0.73.

#### 2.3.4 Infection prevention behaviors

A tool developed by Park et al. (29) was used based on the COVID-19 response guidelines published by the Korea Disease Control and Prevention Agency (30). The tool consists of 13 items designed to assess the level of compliance with the following behaviors: avoiding private gatherings, using public transportation, visiting areas and institutions with infectious diseases, disinfection and ventilation, using hand sanitizers, observing cough etiquette, and wearing a mask. Each item is rated on a 5-point Likert scale, with higher total scores indicating greater compliance with infection prevention behaviors. The reliability of the tool was Cronbach’s α = 0.763 at the time of development; in this study, Cronbach’s α was 0.89. In addition, the Kaiser–Meyer–Olkin values for the e-health literacy scale (eHEALS), risk perception scale, and infection prevention behavior scale all exceeded 0.70, and Bartlett’s tests of sphericity yielded significant results (*p* < 0.001), indicating the adequacy of the sample for factor verification.

### 2.4 Data analysis methods

Statistical analysis was performed using IBM SPSS Statistics for Windows (version 28.0, IBM Corp., Armonk, NY, United States) and PROCESS Macro v4.0 (34). First, frequency analysis was conducted to identify the general characteristics of the study population. Second, a descriptive statistical analysis was conducted to identify the level of e-health literacy, infectious disease risk perception, and infectious disease prevention behaviors of young adults in this study. Third, Pearson’s correlation analysis was conducted to identify the correlations between e-health literacy, infectious disease risk perception, and infectious disease prevention behaviors among young adults. Fourth, independent sample t-tests and one-way analysis of variance (ANOVA) were conducted to verify whether there were significant differences in e-health literacy, infectious disease risk perception, and infectious disease prevention behaviors according to the general characteristics of young adults, and Scheffé’s *post-hoc* test was conducted for variables that showed significant differences.

Fifth, to determine the relationship between e-health literacy, infectious disease risk perception, and infectious disease prevention behavior among young adults and to test whether infectious disease risk perception plays a mediating role in influencing infectious disease prevention behavior, the bootstrapping test proposed by Hayes (34) was conducted. Model 4, the mediation effect model of the process macro, was applied. The number of bootstrap samples was 5,000, and the confidence level was 95% to verify statistical significance.

## 3 Results

### 3.1 General characteristics of young adults

Overall, 224 individuals who provided valid responses to the self-administered questionnaires were included in the analysis (Table 1). By sex, 66 (29.5%) participants were men and 158 (70.5%) were women. By age group, 93 (41.5%) participants were aged <30 years, and 131 (58.5%) were aged ≥30 years. Regarding marital status, 82 (36.6%) were married, 138 (61.6%) were single, and four (1.8%) reported other statuses. With respect to education, 52 (23.2%) were high school graduates, 153 (68.3%) were college graduates, and 19 (8.5%) had graduate education or higher. In terms of employment, 180 (80.4%) were employed and 44 (19.6%) were not. Perceived economic status was reported to be high in 19 (8.5%), medium in 178 (79.5%), and low in 27 (12.1%).

**Table 1:** General participant characteristics.

Variables	Categories	*n* (%)
Sex	Male	66 (29.5)
	Female	158 (70.5)
Age, years	<30	93 (41.5)
	≥30	131 (58.5)
Marital status	Married	82 (36.6)
	Unmarried	138 (61.6)
	Divorced/separated	4 (1.8)
Education	≤High school	52 (23.2)
	College university	153 (68.3)
	≥Graduate school	19 (8.5)
Occupation	Employed	180 (80.4)
	Unemployed	44 (19.6)
Economic status	High	19 (8.5)
	Middle	178 (79.5)
	Low	27 (12.1)
Received infection prevention training	Yes	180 (80.4)
	No	44 (19.6)
Source of infectious disease information	Workplace	57 (25.4)
	Public institution	74 (33.0)
	Mass media	119 (53.1)
	Internet	133 (59.4)
	Other	12 (5.4)
Subjective health status	Healthy	213 (95.1)
	Unhealthy	11 (4.9)
History of infectious disease diagnosis	Yes	156 (69.6)
	No	68 (30.4)
History of isolation	Yes	148 (66.1)
	No	76 (33.9)
Household member with chronic disease	Yes	86 (38.4)
	No	138 (61.6)

Regarding infection prevention education, 180 participants (80.4%) reported prior exposure. Sources of infectious disease information were assessed with multiple responses: workplace, 57 (25.4%); public agencies such as national disease control authorities or public health centers, 74 (33.0%); mass media, 119 (53.1%); the Internet, 133 (59.4%); and other sources, 12 (5.4%).

Most respondents rated their subjective health as good (*n* = 213, 95.1%), while 11 (4.9%) reported it as poor. In total, 156 (69.6%) participants were diagnosed with an infectious disease, and 148 (66.1%) had been quarantined due to an infectious disease. Furthermore, 86 (38.4%) participants reported living with a family member with a chronic disease.

### 3.2 Correlations among e-health literacy, infectious-disease risk perception, and preventive behaviors

Descriptive analyses were conducted to assess the levels of e-health literacy, infectious disease risk perception, and preventive behaviors among young adults (Table 2). All variables were measured on a 5-point scale (1–5). The mean scores for e-health literacy, risk perception were 3.09, and preventive behaviors were 3.55, 3.09, and 3.99, respectively. As all means were ≥3, these constructs can be characterized as moderate to high among the study participants.

**Table 2:** Descriptive statistics and correlation of variables.

Variables	*M* ± standard deviation	1	2	3
		r(p)	r(p)	r(p)
1. eHealth literacy	3.55 ± 0.81	1		
2. Risk perception of infectious disease	3.09 ± 0.49	0.22 (<0.001)	1	
3. Infection prevention behaviors	3.99 ± 0.57	0.22 (<0.001)	0.40 (<0.001)	1

Pearson’s correlation analysis revealed significant positive associations among the three variables. E-health literacy positively correlated with infectious disease risk perception (*r* = 0.22, *p* < 0.001) and preventive behaviors (*r* = 0.22, *p* < 0.001). Infectious disease risk perception also positively correlated with preventive behaviors (*r* = 0.40, *p* < 0.001).

### 3.3 Differences in e-health literacy, infectious disease risk perception, and preventive behaviors by general characteristics

Independent-samples *t*-tests and one-way ANOVA were conducted to examine differences in e-health literacy, infectious-disease risk perception, and preventive behaviors across general characteristics, and Scheffé *post-hoc* tests were applied where appropriate (Table 3). All regression assumptions—normality, homoscedasticity, linearity, and absence of multicollinearity—were satisfied.

**Table 3:** Difference in variables according to general characteristics.

Variables	Categories	Infection prevention behaviors	
		*M* ± standard deviation	*t*/*F* (*p*)
Sex	Male	3.83 ± 0.57	−2.83 (0.005)
	Female	4.06 ± 0.56	
Age, years	<30	4.04 ± 0.50	1.06 (0.292)
	≥30	3.96 ± 0.61	
Marital status	Married	3.99 ± 0.58	1.18 (0.309)
	Unmarried	3.98 ± 0.56	
	Divorced/separated	4.42 ± 0.38	
Education	≤High school	4.09 ± 0.54	1.02 (0.363)
	College university	3.96 ± 0.59	
	≥Graduate school	3.98 ± 0.49	
Occupation	Employed	3.94 ± 0.59	−2.47 (0.014)
	Unemployed	4.18 ± 0.42	
Economic status	High	3.87 ± 0.83	0.68 (0.508)
	Middle	4.01 ± 0.55	
	Low	3.93 ± 0.51	
Received infection prevention training	Yes	4.02 ± 0.56	1.58 (0.115)
	No	3.87 ± 0.60	
Source of infectious disease information	Workplace	4.01 ± 0.55	0.35 (0.724)
	Public institution	4.06 ± 0.63	1.33 (0.185)
	Mass media	4.03 ± 0.58	1.10 (0.272)
	Internet	4.02 ± 0.57	1.08 (0.282)
	Other	3.93 ± 0.56	−0.38 (0.704)
Subjective health status	Healthy	4.00 ± 0.57	1.28 (0.202)
	Unhealthy	3.78 ± 0.49	
History of infectious disease diagnosis	Yes	4.07 ± 0.47	2.69 (0.008)
	No	3.81 ± 0.72	
History of isolation	Yes	4.06 ± 0.54	2.44 (0.016)
	No	3.86 ± 0.61	
Household member with chronic disease	Yes	3.90 ± 0.63	−1.97 (0.051)
	No	4.05 ± 0.52	

Preventive behaviors differed significantly by sex (*t* = −2.83, *p* = 0.005), employment status (*t* = −2.47, *p* = 0.014), prior diagnosis of an infectious disease (*t* = 2.69, *p* = 0.008), and quarantine experience (*t* = 2.44, *p* = 0.016). Specifically, women, unemployed participants, those with prior infectious diseases, and those who had experienced quarantine reported more preventive behaviors than men, employed participants, those without a history of infectious diseases, and those who had not experienced quarantine, respectively.

### 3.4 Relationship between e-health literacy and preventive behaviors, and the mediating role of infectious-disease risk perception

To examine the associations among e-health literacy, infectious disease risk perception, and preventive behaviors, and to explore whether risk perception statistically mediates the association between e-health literacy and preventive behaviors, a bootstrap mediation analysis was conducted as proposed by Hayes (34) (Table 4). The PROCESS macro (Model 4) was applied with 5,000 bootstrap samples and 95% confidence intervals (CIs). Variables that showed significant group differences in prior analyses (sex, employment status, prior diagnosis of infectious disease, and quarantine experience) were entered as covariates.

**Table 4:** Influence relationships among variables.

Path	*B*	SE	*t*	*p*	95% confidence interval	*R*^2^ (adj *R*^2^)
eHealth literacy (X) → Risk perception of infectious disease (M)	0.120	0.042	2.826	0.005	0.036 ~ 0.203	0.136 (0.095)
eHealth literacy (X) → Infection prevention behaviors (Y)	0.103	0.050	2.058	0.041	0.004 ~ 0.201	0.125 (0.084)
eHealth literacy (X) → Infection prevention behaviors (Y)	0.055	0.048	1.142	0.255	−0.040 ~ 0.149	0.227 (0.187)
Risk perception of infectious disease (M) → Infection prevention behaviors (Y)	0.401	0.076	5.274	0.000	0.251 ~ 0.551	

Adjusted for sex, occupation, economic status, infection prevention training, source of infectious disease information, subjective health status, history of infectious disease diagnosis, and isolation.

First, e-health literacy showed a significant positive association with risk perception (*B* = 0.120, *p* = 0.005); this indicated that higher e-health literacy was associated with higher perceived risk.

Second, the overall association between e-health literacy and preventive behaviors was significant (*B* = 0.103, *p* = 0.041).

Third, when e-health literacy and risk perception were entered simultaneously, the direct association between e-health literacy and preventive behaviors was not statistically significant (*B* = 0.055, *p* = 0.255), whereas risk perception demonstrated a significant positive association with preventive behaviors (*B* = 0.401, *p* < 0.001). This pattern suggested that e-health literacy may be associated with preventive behaviors through differences in risk perception, rather than implying a temporal or causal pathway.

Based on the estimated path coefficients, the direct effect of e-health literacy on infectious-disease preventive behaviors was not statistically significant (*B* = 0.055, 95% CI = −0.040 to 0.149) (Table 5). In contrast, the indirect effect of e-health literacy on preventive behaviors via infectious-disease risk perception was significant, as the 95% bootstrap CI did not include zero (*B* = 0.048, 95% CI = 0.016–0.086). These findings indicated that higher e-health literacy was associated with greater risk perception, and that these variables co-occurred with higher levels of compliance with preventive behaviors, without suggesting any temporal relationship.

**Figure 2:**
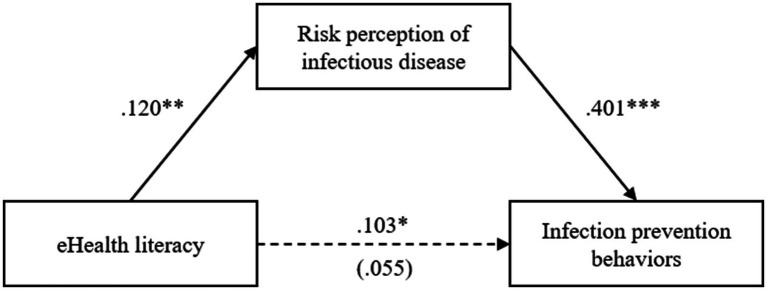
Influence relationships among variables. *p < 0.05; **p < 0.01; ***p < 0.001.

Together, these results support a statistical mediation pattern, which should be interpreted as an associative pathway in this cross-sectional design (Figure 2).

**Table 5:** Direct and indirect effects of eHealth literacy on infection prevention behaviors.

Path	*B*	Standard error	95% confidence interval	
			LLCI	ULCI
Total effect (X → Y)	0.103	0.050	0.004	0.201
Direct effect (X → Y)	0.055	0.048	−0.040	0.149
Indirect effect (X → M → Y)	0.048	0.018	0.016	0.086

LLCI, lower level confidence interval; ULCI, upper level confidence interval.

## 4 Discussion

This study assessed the levels of e-health literacy, infectious disease risk perception, and preventive behaviors among young adults; examined the associations among these variables; and determined the statistical, rather than causal, mediating role of risk perception in the relationship between e-health literacy and preventive behaviors. The major findings of this study are described below.

Pearson correlation analysis demonstrated significant positive associations between e-health literacy, infectious disease risk perception, and preventive behaviors. These findings are consistent with those of previous studies, suggesting that the ability to comprehend health information and risk perception are critical antecedents of infection prevention behaviors (4, 12). In particular, given that seeking health information through digital media has become routine among young adults, higher e-health literacy may increase the likelihood of selecting reliable information and applying it to preventive practices.

Furthermore, the finding that risk perception may serve as a statistical mediating or reinforcing factor, rather than a causal mechanism, aligns with the IMB model (13). Specifically, the results of this study support the theoretical premise that individuals who are more motivated to recognize the severity of and their vulnerability to infectious diseases are more likely to engage in preventive behaviors.

Significant differences in infection prevention behaviors were observed in terms of sex, employment status, prior diagnosis of an infectious disease, and quarantine experience. In this study, young women exhibited significantly higher levels of compliance with infection prevention behaviors than did young men. This finding is consistent with prior evidence that sex is an important determinant of health behaviors (35, 36). Women are generally more proactive in seeking health and public health information and are more likely to engage in health-protective behaviors, such as infection prevention. During the COVID-19 pandemic, women adhere more strictly than men to recommended behaviors, including wearing masks, hand hygiene, and physical distancing (37). These patterns may be related to stronger risk-avoidance tendencies, caregiving roles, and greater sensitivity to public health messaging among women.

Compliance with infection prevention behaviors was also significantly greater among those with a prior diagnosis of an infectious disease than among those without such a history. This suggests that experience of an infection may be related to higher perceived risk and greater engagement in preventive practices, although the temporal direction cannot be established. This is in accordance with the theoretical premise of the IMB model, whereby information and motivation derived from experience, together with behavioral skills, facilitate behavioral change. These results also imply that prevention policies for young adults without prior infection experience should incorporate educational strategies that provide vicarious experiences.

In this study, compliance with infection prevention behaviors was significantly greater among young adults who had been quarantined than among those who had not. This suggests that experience of quarantine was associated with higher risk perceptions and greater adherence, although it does not establish a temporal or causal direction. Quarantine does not merely entail restrictions on physical activity and is often accompanied by social isolation, psychological distress, and academic or occupational disadvantages (38). Such experiences are likely to heighten the perceived seriousness of infectious diseases and, in turn, motivate stricter compliance with preventive behaviors such as hand hygiene, mask-wearing, and physical distancing. Similarly, individuals who have experienced quarantine or self-isolation exhibit higher intentions to comply with public health guidelines (25). Accordingly, prevention policies should incorporate educational approaches that provide vicarious experiences to populations without prior quarantine experience.

In this study, compliance with infection prevention behaviors was significantly greater among young adults who had been quarantined than among those who had not. This suggests that direct experience of quarantine during an outbreak strengthens individual risk perceptions and subsequently promotes adherence to preventive practices. Quarantine does not merely entail restrictions on physical activity but is often accompanied by social isolation, psychological distress, and academic or occupational disadvantages (38). Such experiences likely heighten the perceived seriousness of infectious diseases and, in turn, motivate stricter compliance with preventive behaviors, such as hand hygiene, mask-wearing, and physical distancing. Similarly, individuals who have undergone quarantine or self-isolation exhibit higher intentions to comply with public health guidelines (25). Accordingly, prevention policies should incorporate educational approaches that provide vicarious experiences to populations without prior quarantine experience.

In this study, e-health literacy had a significant positive effect on infectious disease risk perception; young adults with higher e-health literacy tended to perceive greater severity and susceptibility. This finding indicates that e-health literacy extends beyond the ability to search for and understand health information, to include the cognitive processes of evaluating information credibility and interpreting risks (19). Per previous research, individuals with higher e-health literacy are better able to filter misinformation and recognize evidence-based content, leading to more accurate risk appraisals (20, 21). Thus, the present results suggest that enhancing e-health literacy is a crucial strategy not only for improving information use skills but also for shaping appropriate risk perception. This underscores the need to strengthen digital information use training and information verification education in future infection prevention programs.

Furthermore, e-health literacy positively associated with infection prevention behaviors; higher levels of e-health literacy among young adults corresponded to greater adherence to preventive practices. This indicates that e-health literacy functions not only as the capacity to locate and comprehend health information, but also as a determinant that facilitates the translation of information into actionable behaviors (19). It should be noted, however, that the observed correlation coefficient (*r* = 0.22), while statistically significant, reflected a small effect size, suggesting that the individual impact of e-health literacy may be limited (39). This underscores the need to consider broader psychosocial factors—such as social norms, emotional responses, and contextual influences—to more comprehensively understand and enhance preventive behaviors (40). Nevertheless, consistent with previous studies, individuals with higher e-health literacy more actively practiced recommended behaviors such as hand hygiene, mask-wearing, and physical distancing during infectious disease outbreaks (21, 22). These findings support the notion that the ability to identify trustworthy online information and apply it to self-management strategies is directly linked to compliance with infection prevention behaviors. Accordingly, improving e-health literacy should be a central component in designing intervention strategies for infection prevention.

When e-health literacy and infectious disease risk perception were entered simultaneously, the direct association between e-health literacy and preventive behaviors was not statistically significant. In contrast, risk perception showed a significant positive association with infection prevention behaviors, indicating that higher risk perception corresponds to higher levels of preventive practice. Similar results have been reported in prior studies. Patil et al. (21) found that among college students, e-health literacy did not directly influence preventive behaviors, whereas higher perceived risk was associated with increased preventive practices. Similarly, Song and Park (39) reported a strong association between risk perception and compliance with infection prevention behaviors in the early phase of the COVID-19 pandemic in the United States. Given the cross-sectional nature of this study, these associations should not be interpreted as causal associations but as co-occurring behavioral and perceptual patterns.

In this study, e-health literacy did not show a significant direct association with preventive behaviors; however, it showed a significant indirect association via risk perception, and this indicates statistical mediation. This result is consistent with that achieved with the IMB model proposed by Fisher and Fisher (13), which posits that health behavior cannot be explained by information acquisition alone; motivational and psychological factors must act in concert to result in overt actions. In this study, e-health literacy appeared to enhance the ability of individuals to access and use infection-related information. However, the association between information-related factors and preventive behaviors appeared to be statistically mediated by risk perception, without an implication of temporal ordering.

Previous research has emphasized the importance of risk perception for implementation of health behaviors (40, 41). Brewer et al. (40) reported that engagement in infection prevention behaviors such as vaccination was more closely related to perceived risk than was knowledge alone. Similarly, domestic studies conducted during the COVID-19 pandemic identified risk perception as a key driver of preventive action (41). Accordingly, the present findings provide empirical evidence that informational factors such as e-health literacy are statistically related to preventive behaviors through cognitive appraisals, although this pattern does not imply temporal or causal necessity.

From a practical standpoint, these results suggest that the design of education and intervention programs for infection prevention should extend beyond mere promotion of access to and understanding of online health information; in addition, it should strengthen individual perceptions of disease severity and susceptibility using evidence-based, nonfear-arousing approaches. In other words, when strategies to enhance e-health literacy are combined with tailored risk communication that appropriately enhances risk perception, infection prevention behaviors may be promoted more effectively. However, given the cross-sectional design, the temporal sequence of these associations cannot be confirmed, and longitudinal or experimental research is needed to clarify potential causal pathways.

### 4.1 Implications

By empirically delineating the pathways through which e-health literacy and infectious disease risk perception influence preventive behaviors in young adults, this study provides foundational evidence for prevention policies and educational strategies. This evidence confirms the need to integrate the enhancement of e-health literacy with an increase in risk perception.

By identifying differences according to general characteristics, this study highlights the need for tailored intervention strategies based on sex, employment status, economic status, and prior diagnosis/quarantine experience. For example, men, employed individuals, and those without prior infection or quarantine may exhibit lower risk perception and lower levels of preventive behaviors and thus warrant priority for intervention.

These findings are applicable to university and community health-education settings. Beyond simple knowledge transmission, integrative instructional strategies that combine digital information use training with programs to strengthen risk perception are likely to be effective in promoting preventive behaviors among young adults.

### 4.2 Limitations and suggestions for future research

This was a cross-sectional study; therefore, it could not establish clear causal relationships among e-health literacy, risk perception, and preventive behaviors. Longitudinal or experimental studies are needed to confirm these causal pathways. In addition, reliance on self-reported survey data may have introduced response biases—participants’ reported behaviors may not have reflected their actual behaviors and could be influenced by social desirability. This potential bias should be considered when interpreting the results, and future research should enhance validity by supplementing self-reported data with objective measures (e.g., behavioral observations, mobile health records, wearable device metrics). Furthermore, there was a temporal gap between development of the measurement instruments (during the COVID-19 pandemic, 2020–2021) and data collection (post-pandemic, 2025). As the acute threat situation transitioned to a normalized environment by 2025, this delay may have attenuated the risk perceptions and preventive behaviors of participants and reduced the sensitivity of the instruments. Accordingly, future studies should revalidate these instruments in nonpandemic settings or develop updated measures that reflect current infectious disease threats. Moreover, the use of snowball sampling via online communities and social media—while convenient for recruitment—was a nonprobability method that likely introduced sample bias by encouraging participation from individuals with similar characteristics, thereby limiting the representativeness of the sample and the generalizability of the findings. Finally, although this study focused on individual-level factors, actual preventive behavior is shaped by multilevel influences; therefore, future research should examine comprehensive models that integrate individual, interpersonal, and societal determinants.

## 5 Conclusions and recommendations

This study investigated the relationships between e-health literacy, infectious disease risk perception, and infection prevention behaviors in young adults and verified the mediating role of risk perception in the association between e-health literacy and preventive behaviors. The findings were as follows: First, statistically significant positive correlations were found among e-health literacy, risk perception, and infection prevention behaviors. Second, infection prevention behaviors differed significantly by general characteristics, such as sex, employment status, prior diagnosis of an infectious disease, and quarantine experience. Third, e-health literacy had a positive effect on risk perception, and risk perception had a positive effect on preventive behavior performance. Finally, although e-health literacy did not exert a significant effect on preventive behaviors, it had a significant indirect effect on risk perception, indicating that risk perception served as a full mediator.

Accordingly, we offer the following recommendations:

First, when developing educational and intervention programs for infection prevention, an integrated approach that simultaneously strengthens e-health literacy and risk perception is required. Beyond knowledge transfer models, programs should include online health information search and verification training, together with case-based and experiential learning that makes disease severity and susceptibility salient.

Second, tailored interventions should be designed in accordance with differences in general characteristics. In particular, groups that may show relatively lower levels of preventive behaviors—men, employed individuals, those with lower self-rated health, and those without prior diagnosis or quarantine—should be prioritized for targeted education and policy support.

Third, public agencies should enhance access to trustworthy infectious disease information and pair it with media literacy education so that young adults can identify misinformation in digital environments.

Fourth, future research should address the limitations of the present study by adopting a longitudinal design to verify causal relationships and incorporating objective indicators, such as behavioral observations, mobile health data, and wearable devices, to overcome the constraints of self-reporting. Additionally, expanding the sample to include diverse regions and cultural contexts would improve the generalizability of the findings.

## Data Availability

The original contributions presented in the study are included in the article/supplementary material, further inquiries can be directed to the corresponding author.
